# Mitochondrial type II NAD(P)H dehydrogenases in fungal cell death

**DOI:** 10.15698/mic2015.03.192

**Published:** 2015-03-02

**Authors:** A. Pedro Gonçalves, Arnaldo Videira

**Affiliations:** 1 ICBAS-Instituto de Ciências Biomédicas de Abel Salazar, Universidade do Porto, Rua de Jorge Viterbo Ferreira 228, 4050-313 Porto, Portugal.; 2 IBMC-Instituto de Biologia Molecular e Celular - Universidade do Porto, Rua do Campo Alegre 823, 4150-180 Porto, Portugal.; 3 Instituto de Investigação e Inovação em Saúde, Universidade do Porto, Portugal.; 4 Current address: Plant and Microbial Biology Department, The University of California, Berkeley, CA 94720, USA.

**Keywords:** alternative NAD(P)H dehydrogenases, fungi, programmed cell death, ROS

## Abstract

During aerobic respiration, cells produce energy through oxidative phosphorylation, which includes a specialized group of multi-subunit complexes in the inner mitochondrial membrane known as the electron transport chain. However, this canonical pathway is branched into single polypeptide alternative routes in some fungi, plants, protists and bacteria. They confer metabolic plasticity, allowing cells to adapt to different environmental conditions and stresses. Type II NAD(P)H dehydrogenases (also called alternative NAD(P)H dehydrogenases) are non-proton pumping enzymes that bypass complex I. Recent evidence points to the involvement of fungal alternative NAD(P)H dehydrogenases in the process of programmed cell death, in addition to their action as overflow systems upon oxidative stress. Consistent with this, alternative NAD(P)H dehydrogenases are phylogenetically related to cell death - promoting proteins of the apoptosis-inducing factor (AIF)-family.

## INTRODUCTION

**Figure 1:**
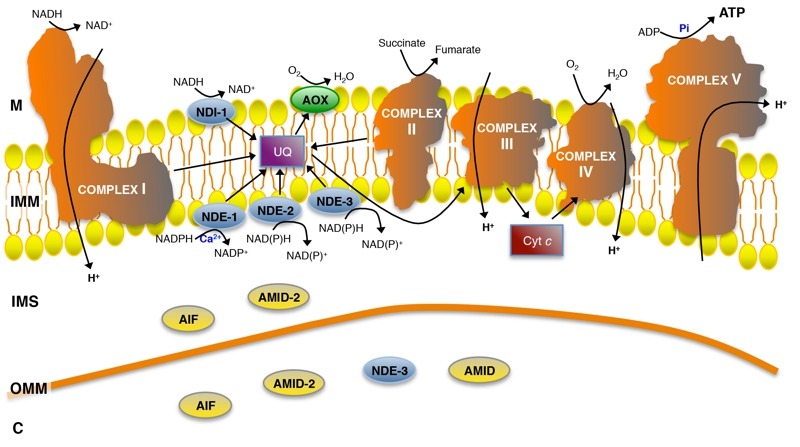
FIGURE 1: Representation of the mitochondrial respiratory chain, alternative NAD(P)H dehydrogenases, alternative oxidase systems and AIF-family proteins of *N. crassa*. M: mitochondrial matrix; IMM: mitochondrial inner membrane; IMS: intermembrane space; OMM: mitochondrial outer membrane; C: cytosol; UQ: ubiquinone; Cyt *c*: cytochrome *c*.

Mitochondria (from the Greek *mitos *and *chondros*, meaning "thread" and "granule", respectively) are the dynamos of the eukaryotic cell due to their major role in energy production under aerobic conditions. They are double membrane organelles: the protein-rich core of the organelle is known as the matrix, whereas the inner and outer mitochondrial membranes (IMM and OMM, respectively) delimitate the intermembrane space. The inner membrane forms a series of invaginations designated as *cristae*. Mitochondria take up pyruvate formed during the first stage of carbon metabolism (glycolysis) and fatty acids, and convert them into energy 1. The respiratory chain in the IMM is composed of the multi-subunit enzymatic complexes (complex I, II, III and IV), together with ubiquinone (coenzyme Q) and cytochrome *c* (Fig. 1). These complexes possess a number of protein-associated prosthetic groups - flavin mononucleotide (FMN), flavin adenine dinucleotide (FAD), iron-sulfur clusters (FeS), iron and copper ions and heme - that transport electrons. Ubiquinone and cytochrome *c* transfer electrons between complexes. The electrochemical gradient that triggers the rotation of the ATP synthase (complex V), which leads to the formation of ATP from the phosphorylation of ADP 2,3, is generated by the proton-pumping activity of (i) complex I (NADH:ubiquinone oxidoreductase), which uses NADH as a source of electrons, transferring them to ubiquinone via FMN and a series of iron-sulfur clusters, (ii) complex III (ubiquinol cytochrome *c* reductase), which transfers electrons from the reduced ubiquinone or ubiquinol to cytochrome *c*, and (iii) complex IV (cytochrome *c* oxidase), which catalyses electron transfer to molecular oxygen and reduces it to water. Complex II (succinate dehydrogenase) transfers electrons from succinate to ubiquinone, providing an alternative electron entry point into the respiratory chain without proton pumping. Apart from the generation of energy, mitochondria are involved in several other cellular processes, like the biogenesis of iron-sulphur clusters, Ca^2+^ storage, intermediary metabolism, coenzyme biosynthesis and cell death 1.

## BRANCHED RESPIRATORY SYSTEMS IN FUNGI

In some fungi, plants, protists and bacteria, the electron transport chain is branched into single polypeptide alternative systems with no proton translocation activity that bypass the canonical pathway. The cyanide-resistant alternative oxidase (AOX) constitutes a well-established bypass of

complexes III and IV, whereas type II NAD(P)H dehydrogenases (or alternative NAD(P)H dehydrogenases) bypass complex I 4,5. Alternative NAD(P)H dehydrogenases are particularly important not only because they oxidize NAD(P)H and reduce quinones but also because they serve as entry points for electrons into the respiratory chain 6,7. Their importance is firmly demonstrated in *Saccharomyces cerevisiae*, where complex I is absent 8 and type II NAD(P)H dehydrogenases are the only existing NAD(P)H oxidases 9,10. These enzymes are flavoproteins resistant to classical complex I inhibitors, such as rotenone or piericidin A, and there is no selective and reliable drug to block them so far, although inhibition is feasible with diphenyleneiodonium 11,12,13. Alternative NAD(P)H dehydrogenases usually, but not always, contain FAD as the sole prosthetic group 4,5,7. Recently, an *in silico* approach identified putative alternative NAD(P)H dehydrogenases in a few metazoan organisms, but a functional verification is still missing 14.

In addition to complex I, our group characterized four alternative rotenone-insensitive NAD(P)H dehydrogenases in *Neurospora crassa* (Fig. 1 and Table 1) 6,7. They are associated with the inner mitochondrial membrane, but while one of them, NDI-1 15, is localized at the matrix side of the membrane, the other three, NDE-1 16,17, NDE-2 18 and NDE-3 19, are facing the intermembrane space. Interestingly, NDE-3 was also found in the cytosol 19.

**Table 1:** Main features of *N. crassa* alternative NAD(P)H dehydrogenases. ^a^ Ca^2+^ stimulates the oxidation of cytosolic NADH in a Δ*nde-1*Δ*nde-2* double mutant, but not in the triple mutant Δ*nde-1*Δ*nde-2*Δ*ndi-1*, indicating that NDI-1 may be stimulated by Ca^2+^ 18. NA: not assessed.

**Protein**	**Topology**	**Substrate specificity**	**Ca^2+^**	**pH**	**Reference(s)**
NDE-1	External	Cytosolic NADPH	Ca^2+^-dependent	Physiological pH	16,17
NDE-2	External	Cytosolic NADH and NADPH	-	NADH throughout the pH range and NADPH at acidic pH	18
NDE-3	External	Cytosolic NADH and NADPH	-	NA	19
NDI-1	Internal	Matrix NADH	Ca^2+^-stimulated? ^a^	NA	15,18

Plants contain even more type II NAD(P)H dehydrogenases than fungi. Seven of these enzymes have been identified in *Arabidopsis thaliana*: three external (NDB1, NDB2 and NDB4), three internal (NDA1, NDA2 and NDC1) and one uncharacterized (NDB3) 20,21,22. Motifs in the N’-terminal portion of the proteins appear to determine mitochondrial import and their localization to either side of the inner membrane 23. Interestingly, dual targeting to mitochondria and chloroplasts or peroxisomes was claimed in some cases, although its functional relevance is unknown 24.

Alternative NAD(P)H dehydrogenases may be organized in supramolecular entities, similarly to the respiratory chain supercomplexes. There is evidence showing that in yeast these enzymes form a complex with a glycerol-3-phosphate dehydrogenase, two L-lactate-dehydrogenases, a few enzymes from the tricarboxylic acid cycle, two probable flavoproteins and an acetaldehyde dehydrogenase 25. In addition, the *Yarrowia lipolytica* alternative external NADH dehydrogenase and complex IV are associated, particularly in high energy-requiring, logarithmic-growth phase cells 26,27. Current literature suggests that the formation of supercomplexes, that include NAD(P)H dehydrogenases, might be related with electron channelling 25,27. In *N. crassa*, there is no evidence for the formation of supercomplexes containing alternative NAD(P)H dehydrogenases 28, but observations point to some kind of interaction between NDE-2 and complex I 18. *N. crassa* NDE-1 stands out because of its unique NADPH selectivity and regulation by pH and Ca^2+^ 17, the latter feature likely related to the presence of a conserved Ca^2+^-binding domain 16. In plants, the external NDB1 oxidizes NADPH in a Ca^2+^-dependent manner while NDB2 is a NADH dehydrogenase stimulated by Ca^2+^ 22,29.

The physiological role of alternative NAD(P)H dehydrogenases is still somehow controversial, although it is fairly well established that they confer metabolic plasticity allowing cells to adapt to different environmental and stress conditions. They may act as overflow systems keeping cytosolic and mitochondrial reducing equivalents (NADH, NADPH) at physiological levels, thus avoiding potential tricarboxylic cycle repression by elevated NADH levels and excessive levels of reactive oxygen species (ROS) 4,5,7,30. Heterologous expression of Ndi1 from yeast was shown to reduce mammalian complex I-mediated ROS generation 31. In contrast, *S. cerevisiae* alternative NADH dehydrogenases have been proposed as potential sources of superoxide radicals by other studies 32,33,34.

In *N. crassa*, expression of alternative NAD(P)H dehydrogenases genes greatly depends on the growth phase 15,18,19,35. It is not possible to obtain viable double mutants between NDE-2 and complex I mutants that lack a functional enzyme, suggesting that NDE-2 and complex I interact in a yet unidentified pathway 18.

Humans do not possess alternative NAD(P)H dehydrogenases, but enzymes from other organisms have potential to be used in gene-based therapies. Heterologous expression of the yeast Ndi1 restores respiration in complex I-deficient human cells 36 and was also shown to be protective in *in vivo *models of Parkinson’s disease 37,38, Leber's hereditary optic neuropathy 39 and breast cancer 40.

Because mammals lack alternative NAD(P)H dehydrogenases, these enzymes are good candidate targets for human therapy in cases of fungal infection. The crystal structure of yeast Ndi1 has been recently solved and will allow a better understanding of the regulatory mechanisms of type II NAD(P)H dehydrogenases and likely lead to an evaluation of their potential as therapeutical agents or targets 41,42.

## ALTERNATIVE NAD(P)H DEHYDROGENASES AS MEDIATORS OF PROGRAMMED CELL DEATH

Several reports point to a role of fungal alternative NAD(P)H dehydrogenases in cell death. In *S. cerevisiae*, overexpression of the internal Ndi1 (proposed as the yeast homologue of the human apoptosis-inducing factor-homologous mitochondrion-associated inducer of death or AMID), but not of the external Nde1, leads to ROS-mediated apoptosis-like cell death, particularly in glucose-rich media. The authors showed that the disruption of both of these NADH dehydrogenases results in lower ROS production and increased chronological life span accompanied by reduced fitness 34.

More recently, yeast Ndi1 was also shown to be involved in cell death induced by different stimuli like hydrogen peroxide, acetic acid and manganese ions, independently of its oxidoreductase activity 43. During the execution of manganese ion-induced cell death, a N’-terminal portion of Ndi1 is cleaved and the protein translocates to the cytoplasm. However, in sharp contrast, it was reported that overexpression of yeast Ndi1 in human cell lines prevents rotenone- and paraquat-induced cell death 44,45.

Thus, the specific role of alternative NAD(P)H dehydrogenases in the protection or enhancement of ROS production is still uncertain 27,31,32,45,46,47,48. Although speculative, it is possible that the discrepant findings observed in human and yeast cells overexpressing Ndi1 result from the fact that on the one hand, human cells do not possess these enzymes, and one the other hand, yeast cells harbor additional NAD(P)H dehydrogenases. In both cases, this suggests that alternative NAD(P)H dehydrogenases may interact with each other or respond to overexpression or downregulation of other members of the family. In fact, compensatory mechanisms of gene expression have been demonstrated in *N. crassa *strains lacking one or more NAD(P)H dehydrogenases (see below).

In *N. crassa*, a double mutant devoid of NDE-1 and NDE-2 displays lower ROS accumulation, increased catalase activity and resistance to paraquat 46. Particularly, NDE-2 appears to be engaged in mitochondrial ROS generation. In *A. nidulans*, the expression of genes encoding NAD(P)H dehydrogenases are induced upon exposure to different cell death stimuli, especially farnesol 49. Moreover, while the overexpression of NdiA augments the resistance to farnesol, the deletion of NdeA results in hypersensitivity to the drug. The latter is likely due to increased accumulation of ROS in the presence of farnesol 49. In *N. crassa*, disruption of *nde-1* leads to increased susceptibility to staurosporine, associated with higher ROS accumulation and altered intracellular Ca^2+^ dynamics (Gonçalves AP, Cordeiro JM, Monteiro J, Lucchi C, Correia-de-Sá P, Videira A, unpublished data). In addition, a yeast *NDE1* deletion strain is more resistant to artemisinin and dimeric naphthoquinones 50,51. Despite the aforementioned controversy around the role of NAD(P)H dehydrogenases during cell death, a current view is that these enzymes seem to be activated in different model organisms in conditions of highly reducing cellular environment, diverging electron transfer from the canonical respiratory chain pathway and thus avoiding system overflow and deleterious ROS production 27,47.

Notably, alternative NAD(P)H dehydrogenases are protein homologues of apoptosis-inducing factor (AIF)-family members, namely the well established cell death executioners AIF and AMID (Fig. 1). AIF-family members have been described as oxidoreductases 52,53, but disruption of AIF or AMID does not affect complex I activity, nor does the supramolecular organization of the respiratory chain in *N. crassa* 35. In this fungus, AIF was found both in mitochondrial and cytosolic extracts 35, while, for comparison, two AIFs localized in the mitochondria and in the cytoplasm, respectively, have been reported in *Podospora anserina* 54. In *N. crassa*, AMID was found exclusively in the cytosol whereas a third member of this family, AMID-2, was found both in the mitochondria and in the cytosol but was only observed when AMID was absent, suggesting overlapping functions 35. A genome-wide association study on a wild population of *N. crassa* showed a genetic interaction between *amid-2* and *czt-1*, a transcription factor that controls cell death and drug resistance 55.

In *N. crassa*, analysis of gene expression profiles of these families of genes in simple or multiple deletion strains for alternative NAD(P)H dehydrogenases showed the occurrence of compensatory mechanisms 19,35,46. For instance, *ndi-1 *is upregulated in a ∆*nde-1*∆*nde-2* and in a ∆*nde-2 *strain, *amid *and *amid-2 *are upregulated in a triple ∆*ndi-1*∆*nde-1*∆*nde-2* mutant, *nde-1 *is upregulated in ∆*nde-3* cells and *nde-2 *is upregulated in ∆*ndi-1*, ∆*nde-1* and ∆*nde-3* single mutants. The functional meaning of this compensation in gene expression is currently unknown, but there is evidence in yeast that mitochondrial dysfunction leads to an alteration in gene expression through retrograde signaling in order to reduce the impact of this dysfunction on cellular fitness 56. Interestingly, a phylogenetic analysis showed that *N. crassa *NDE-3 clusters with AIF-like proteins rather than with the other NAD(P)H dehydrogenases 35, further suggesting a close relationship between these proteins.

Accumulating evidence clearly relates alternative NAD(P)H dehydrogenases to intracellular cell death routes. However, further studies are needed to better understand the mechanisms underlying their involvement in the cellular responses to cell death stimuli.
